# Involvement of immune system and Epithelial–Mesenchymal-Transition in increased invasiveness of clustered circulatory tumor cells in breast cancer

**DOI:** 10.1186/s12920-021-01112-9

**Published:** 2021-11-20

**Authors:** Samane Khoshbakht, Sadegh Azimzadeh Jamalkandi, Ali Masudi-Nejad

**Affiliations:** 1 Laboratory of Systems Biology and Bioinformatics (LBB), Department of Bioinformatics, Kish International Campus, University of Tehran, Kish Island, Iran; 2 Chemical Injuries Research Center, Systems Biology and Poisonings Institute, Tehran, Iran; 3 Laboratory of Systems Biology and Bioinformatics (LBB), Institute of Biochemistry and Biophysics (IBB), University of Tehran, Tehran, Iran

**Keywords:** Breast cancer, Single/cluster CTC, Metastasis, Directed network, Epithelial-mesenchymal transition, Immune response

## Abstract

**Background:**

Circulating tumor cells (CTCs) are the critical initiators of distant metastasis formation. In which, the reciprocal interplay among different metastatic pathways and their metastasis driver genes which promote survival of CTCs is not well introduced using network approaches.

**Methods:**

Here, to investigate the unknown pathways of single/cluster CTCs, the co-expression network was reconstructed, using WGCNA (Weighted Correlation Network Analysis) method. Having used the hierarchical clustering, we detected the Immune-response and EMT subnetworks. The metastatic potential of genes was assessed and validated through the support vector machine (SVM), neural network, and decision tree methods on two external datasets. To identify the active signaling pathways in CTCs, we reconstructed a casual network. The Log-Rank test and Kaplan–Meier curve were applied to detect prognostic gene signatures for distant metastasis-free survival (DMFS). Finally, a predictive model was developed for metastasis risk of patients using VIF-stepwise feature selection.

**Results:**

Our results showed the crosstalk among EMT, the immune system, menstrual cycles, and the stemness pathway in CTCs. In which, fluctuation of menstrual cycles is a new detected pathway in breast cancer CTCs. The reciprocal association between immune responses and EMT was identified in CTCs. The SVM model indicated a high metastatic potential of EMT subnetwork (accuracy, sensitivity, and specificity scores were 87%). The DMFS model was identified to predict patients’ metastasis risks. (c-index = 0.7). Finally, novel metastatic biomarkers of *KRT18* and *KRT19* were detected in breast cancer CTCs.

**Conclusions:**

In conclusion, the reciprocal interplay among critical unknown pathways in CTCs manifests both their survival in blood and metastatic potentials. Such findings may help to develop more precise predictive metastatic-risk models or detect pivotal metastatic biomarkers.

**Supplementary Information:**

The online version contains supplementary material available at 10.1186/s12920-021-01112-9.

## Background

Metastasis is the leading cause of death among women with breast cancer [1, 2]. Cancer progression and metastasis are critical and even controversial aspects of cancer studies [2]. There are two arguable metastasis models, including parallel progression and linear progression, which try to explain the dark side of the tumor developments [3]. In the linear model, the tumor initiates by genetic or epigenetic alternations, grows, spreads, and gains metastasis potentials to disseminate ectopic sites; Contrarily, in the parallel model, the metastasis ability initiates early-onset and evolves by circulating tumor cells (CTCs), parallelly [3, 4]. CTCs, which negatively relate to the high rise of mortality rates in cancer, are rare disseminated tumor cells in the peripheral blood of patients [5]. Of note, they appear even in the early stages and are prominent and leading components in metastasis [2, 6]. Therefore, the detection of CTCs in metastatic and non-metastatic breast cancer patients implies their leading role in cancer progression [7]; Moreover, their physical characteristics as single CTC or CTC clusters play a crucial role in metastasis propensity [5]. They borrow the morphologic features of their primary tumors and gain new features to survive in blood [8]. CTC clusters, which consist of 2–50 cancer cells, can transit through the circulation of patients and increase the potential of metastasis to 23- to 50-fold [5]. They overcome many hurdles to colonize distant organs including intravasation into circulation, evading immune bulwarks, extravasation to distant sites, and eventually replacing the microenvironment of host tissue [9, 10].

Of note, the signaling pathways or intrinsic molecular characteristics of the single/cluster CTCs are not well recognized. Therefore, fully realizing the CTCs' cellular features using network approaches, will guide us to unknown metastasis concepts and more precise therapeutic decisions; In which, the reversal phenotypic of Epithelial–Mesenchymal-Transition (EMT) or immune system are two prominent components in cancer progression [1, 11, 12]. EMT mechanism, which helps cancer cells lose their cell adhesion and gain mesenchymal phenotype, accelerates metastasis through immunosuppression in primary tumors [12–14]. Accordingly, assessment of the role of EMT and immune responses in CTCs as well as intermediate pathways is essential in cancer biology.

In this study, we implemented the co-expression network reconstruction for CTCs isolated from advanced patients’ blood. We extracted metastasis-relevant subnetworks that enriched the immune system and EMT pathways. The preservation of subnetworks was assessed in GSE51827. The metastasis-free survival analysis and Kaplan–Meier curve of genes were implemented in GSE7390 (external data). Concerning a better understanding of signaling pathways inside CTCs, we also extracted a signaling subnetwork from the KEGG database. To determine the metastasis potentials of identified subnetworks, we carried out the SVM, neural network, and decision tree classifications on GSE7390, and the selected model was validated in GSE9195. We also developed a metastasis-free-survival-risk model to predict patients’ risk, using the VIF-stepwise feature selection and cox-PH model. Finally, an article review was implemented to detect novel metastatic biomarkers in breast cancer.

## Methods

### Data sets and metadata information

The single-cell RNA-seq data related to advanced ER + breast cancer patients were downloaded from the NCBI data repository (GSE86978, AB 5500xl Genetic Analyzer). The data consist of 77 cells which 47 of them were CTC clusters, 22 cells were single CTCs, and the rest of the cells were not categorized. This dataset was used to reconstruct the co-expression network and extract subnetworks. The GSE51827 (AB 5500xl Genetic Analyzer), which consists of 29 cells (15 single CTCs and 14 CTC clusters), was implemented to check the preservation of subnetworks in the second dataset.

The gene expression of GSE7390, which consists of 198 untreated breast cancer patients, was used for the assessment of metastasis potential of subnetworks. Moreover, the GSE9195, which consists of 77 breast cancer patients with ER + subtype, was applied for validation of the classification model, overall survival, and metastasis-free survival analyses.

### Quality control, pre-processing, normalization, and differential analysis

The heterogeneity in cancer samples and transcriptomics data is a major concern, particularly in single-cell-based studies. Therefore, to have more precise downstream analyses and remove non-biological variations, we implemented multiple quality control and pre-processing steps on both cells and gene-level. For cell level quality control, low-quality cells were removed to reduce the effect of technical errors using the Scater package in R that is specifically modified for single-cell studies [15]. The calculateQCMetrics and isOutlier functions were used to detect low-quality cells. In this step, three cells out of 77 cells were removed in GSE86978.

For gene-level quality control, genes with zero expression values and genes that had at least one not available value (NA) were filtered out in the pre-processing step. Finally, the expression data were normalized using the Scater package [15]. Additionally, before network reconstruction, we used hierarchical clustering in the WGCNA package to detect outliers in the datasets [16]. Therefore, we conducted downstream analyses with 74 cells for GSE86978 dataset [15]. Moreover, we applied the same steps for GSE51827, but no outlier cells were detected. We should emphasize that the count matrix for GSE86978 and GSE51827 datasets were according to the original publication. Both datasets had the AB 5500xl Genetic Analyzer platform, but our normalization method was different from the original articles. We selected the ER + breast cancer patients. Therefore, 134 out of 198 patients were filtered for GSE7390. All patients in GSE9195 had ER + subtype. Therefore, all patients were included. The expression data of GSE7390 and GSE9195 were normalized using the Robust Multichip Average (RMA) method [17].

The differential expression analysis (DEA), which compares clustered cells' expression to the single cells' expression, was implemented using the limma package in R (FDR < 0.05, Benjamini and Hochberg method) [18].

### Co-expression network (CEN) reconstruction and subnetwork extraction

The co-expression network was reconstructed using a weighted correlation network analysis (WGCNA) method [16]. The pairwise relation among genes was estimated using the Pearson correlation among genes. Concerning having more connected subnetworks, we carried out the topological overlap matrix (TOM) and connectivity gene filtering (connectivity values less than 0.1 were omitted) [16]. Higher connectivity values indicate more considerable co-expressed subnetworks [16, 19]. Eventually, we used hierarchical clustering to extract subnetworks. The trait used in this study was the cluster and single status of the cells captured in blood. The subnetworks, which have strong correlations between their first principle component and the biological trait, were selected as trait-related (metastasis) subnetworks. The gene significance and module membership were used to filter essential genes in selected subnetworks. The gene significance is the correlation between gene expression and the metastasis trait. The module membership is the correlation between gene expression and module representative (first principle component in the principal component analysis (PCA)). The preservation of subnetworks were assessed in the external dataset (GSE51827).

### Signaling network reconstruction

We downloaded all homo sapiens pathways from the Kyoto Encyclopedia of Genes and Genomes (KEGG) database resource and merged them [20]. Furthermore, the KEGG ids were annotated to gene symbols. At the last step, to have casual relations among genes, we extracted a directed induced subnetwork from the KEGG database using two detected significant subnetworks ($\left|\mathrm{correlation}\right|$>0.5 was considered significant) [21]. The genes were categorized based on biological processes (BP of gene ontology) terms using the ClueGO plug-in in Cytoscape [22, 23]. The network visualization was implemented by the Cytoscape and the Gephi software [23, 24].

### Gene set enrichment analysis and subnetwork preservation analysis

The significant trait-related, metastatic, subnetworks ($\left|\mathrm{correlation}\right|$>0.5) were enriched, using ConsensusPathDB webserver (q-value < 0.05) [25]. The GSE51827, which has CTC gene expression, was downloaded from NCBI to implement preservation analysis of subnetworks in the external dataset in R [16, 19, 26]. The scater package, which is suitable for single-cell RNA-seq data, was used to preprocess, normalize, and merge expression data [15].

The combined statistics for preservation assessment, which includes ${Z}_{summary }$ and ${Median}_{rank}$, were used to check the reproducibility of subnetworks [19]. The ${Z}_{summary}$, which shows the interaction pattern among genes in subnetworks, evaluate connectivity and density in the external dataset. ${Z}_{summary }<2$ indicates not preserved subnetworks. If $2<{Z}_{summary}<10$, the subnetwork is semi preserved, and if ${Z}_{summary}>10$, the subnetwork is preserved. Moreover, a higher ${Median}_{rank}$ indicates more preservation of subnetworks in the external dataset [19]. The ${Z}_{summary}$ and ${Median}_{rank}$ were assessed for our subnetworks.

### Assessment of distant metastasis potential of subnetworks

To evaluate the importance of selected subnetworks and the metastasis potential of genes, we implemented the classification algorithms on two individual datasets (GSE7390 and GSE9195). In this section, we learned three classifiers, including support vector machine (SVM), artificial neural network (ANN), and decision tree on metastatic and non-metastatic patients [27]. The classification algorithms were run with and without feature selection algorithms, including the genetic algorithm (GA) and the world competitive contest (WCC) algorithm. The SVM was implemented with fivefold cross-validation, linear kernel, and 80 percent of cells as the training set. Finally, the accuracy, precision, and specificity were checked to select a better classifier for metastasis prediction; Furthermore, to identify the most metastatic-related subnetwork. To assess the reproducibility of our results, the selected model was validated in another dataset (GSE9195).

### Distant metastasis-free survival analysis

The Kaplan–Meier curve, distant metastasis-free survival, and overall survival analyses were implemented using GSE7390 in R [28]. The patients were stratified due to quartiles. The expression values lower than the first quartile were labeled low expression, and expression values higher than the third quartile were labeled high expression. The stepwise Cox proportional hazard ratio (Cox-PH) was implemented for selected subnetworks [28]. The concordance index was calculated to evaluate model performance. The Variance Inflation Factor (VIF) lower than two was used as the variable selection criteria. The first and third quartiles of the predicted hazard ratio were used for stratifying patients into three groups, including low-risk, medium-risk, and high-risk groups.

## Results

### Pre-processing of CTCs and DEA

After the quality control and pre-processing step for GSE86978, 74 out of 77 cells were included in the downstream analyses. The excluded cells revealed low quality, therefore, they were filtered out. The differential expression analysis was implemented after the normalization step (FDR < 0.05) [18]. The adjusted p-values and logarithm of fold changes were reported in Additional files 1 and 2.

**Fig. 1:**
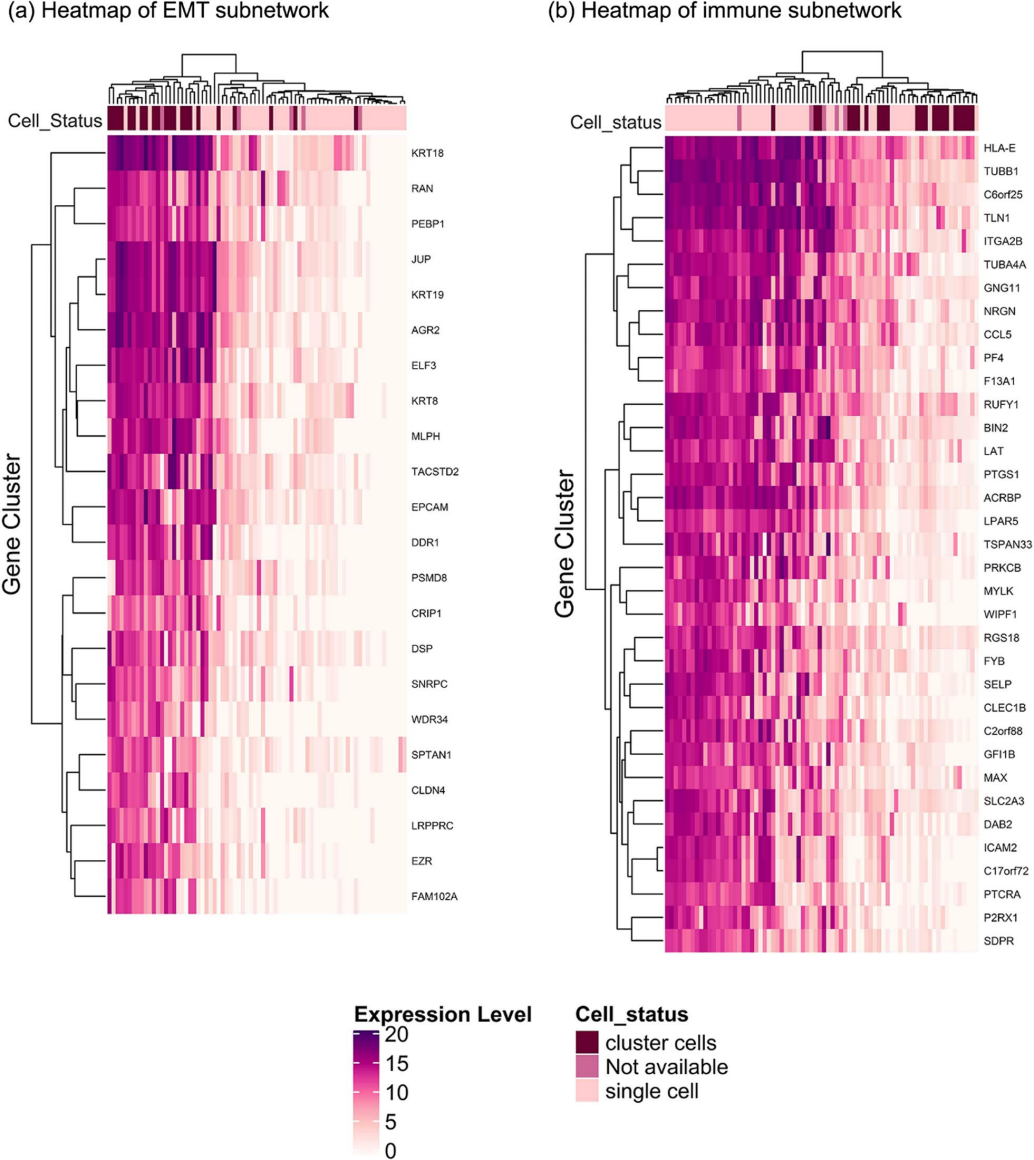
CTC cluster vs. single CTCs gene expression change. **a** Gene expression changes between CTC clusters and single CTCs for EMT-related subnetwork. **b** Gene expression changes between CTC clusters and single CTCs for Immune-related subnetwork

We discovered the differential expression between CTC clusters and single CTCs for two identified subnetworks (EMT and Immune). As shown in Fig. 1, the immune subnetwork genes were downregulated in CTC clusters (light purple color in the heatmap), and the genes of the EMT subnetworks were upregulated (dark purple color in the heatmap) in CTC clusters in comparison with single CTCs. Figure 1 was extracted from the GSE86978 dataset. Moreover, using identified subnetworks, the single and cluster CTCs were grouped well in both subnetworks (Fig. 1a, b). The immune-related subnetwork represented a stronger expression difference between clustered and single cells. Figure 1 was extracted from GSE86978.

### Metastasis associated subnetworks

**Fig. 2:**
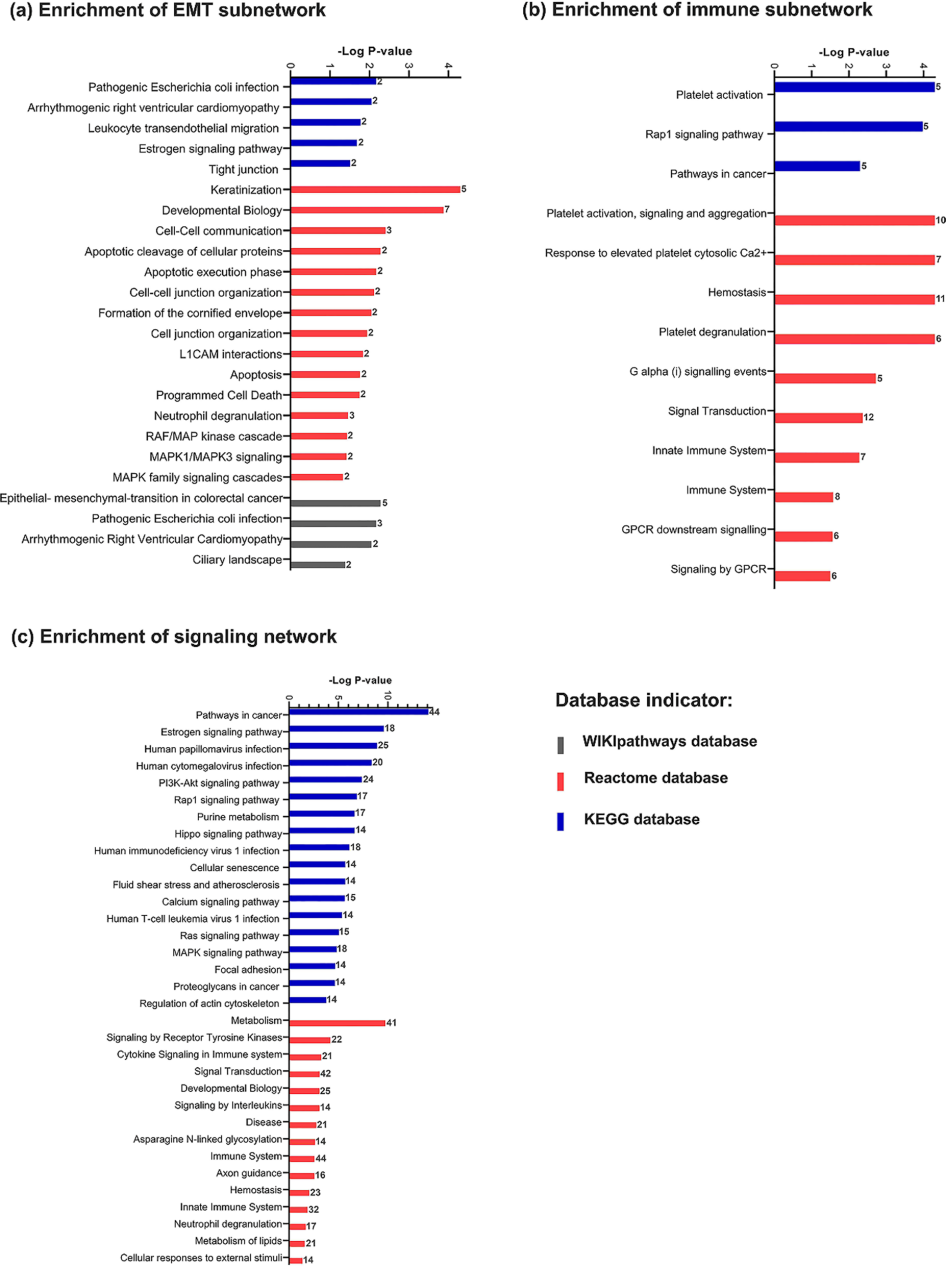
Gene set enrichment analysis. The numbers for each bar indicate the number of significant genes. **a** Significant pathways of EMT subnetwork (size = 22) (q-value < 0.05). **b** Significant pathways of Immune subnetwork (size = 35) (q-value < 0.05). **c** Significant pathways of signaling network of CTCs (q-value < 0.05)

Metastasis-associated subnetworks were determined, using co-expression analysis and hierarchical clustering [16]. We detected 16 subnetworks. The first principle component (in PCA analysis) of subnetworks and the trait (cluster CTCs vs. single CTCs) association was assessed by correlation analysis. The two top significant subnetworks, which had the highest correlation with the trait, were nominated for enrichment analysis ($\left|\mathrm{midnightblue correlation}\right|$=0.57, $\left|\mathrm{turquoise correlation}\right|$= 0.51) (Additional file 3). The sizes of midnightblue and turquoise subnetworks were 35 and 22 genes, and they were enriched for immune responses and EMT pathways, respectively (Fig. 2).

To have a biological concept for subnetworks, we addressed the midnightblue and the turquoise subnetworks, the Immune and EMT subnetworks, respectively, The EMT subnetwork included cancer-related pathways such as ‘cell–cell communication’, ‘tight junction’, ‘keratinization’, and ‘estrogen signaling pathway’. The Immune subnetwork included pathways such as ‘platelet activation’, ‘immune system’, and ‘innate immune system’. After having reviewed the literature, we detected the novel metastatic biomarkers in breast cancer. The immune-related novel metastatic biomarkers in breast cancer were *PTCRA*, *F13A1*, *LAT*, *GNG11*, *ICAM2*, *NRGN*, *P2RX1*, *CLEC1B*, *BIN2*, *LPAR5*, *CCL5*, *SELP*, *RUFY1*, *C6ORF25*, *TUBB1*, *GFI1B*, *C2ORF88*, *ACRBP*, and *C17ORF72*. Module membership and gene significance of the Immune subnetwork were reported in Additional file 1.

The EMT-related genes were *LRPPRC*, *AGR2*, *CLDN4*, *CRIP1*, *DSP*, *ELF3*, *JUP*, *KRT8*, *KRT18*, *KRT19*, *FAM102A*, *TACSTD2*, *EPCAM*, *PEBP1*, *PSMD8*, *RAN*, *SNRPC*, *SPTAN1*, *EZR*, *DDR1*, *MLPH*, and *WDR34*. In which, gene *SNRPC*, upregulated in CTC clusters, is a metastatic novel biomarker in breast cancer. Module membership and gene significance for the EMT subnetwork were summarized in Additional file 2.

The preservation of all subnetworks was assessed in the external dataset (GSE51827). The two combined statistics ${Z}_{summary }$ and ${Median}_{rank}$ were calculated to assess subnetworks preservation in the second dataset (Immune subnetwork: ${Z}_{summary }=14$, ${Median}_{rank}=9$ and EMT subnetwork: ${Z}_{summary }=31$,${Median}_{rank}=6$). The ${Z}_{summary}$ values for both subnetworks were more than 10, and ${Median}_{rank}$ values were low; Subsequently, these statistics indicated the Immune and EMT subnetwork preservations in the second dataset. The statistics for all subnetworks were reported in Additional file 4).

### Detection of crosstalk among pathways

**Fig. 3:**
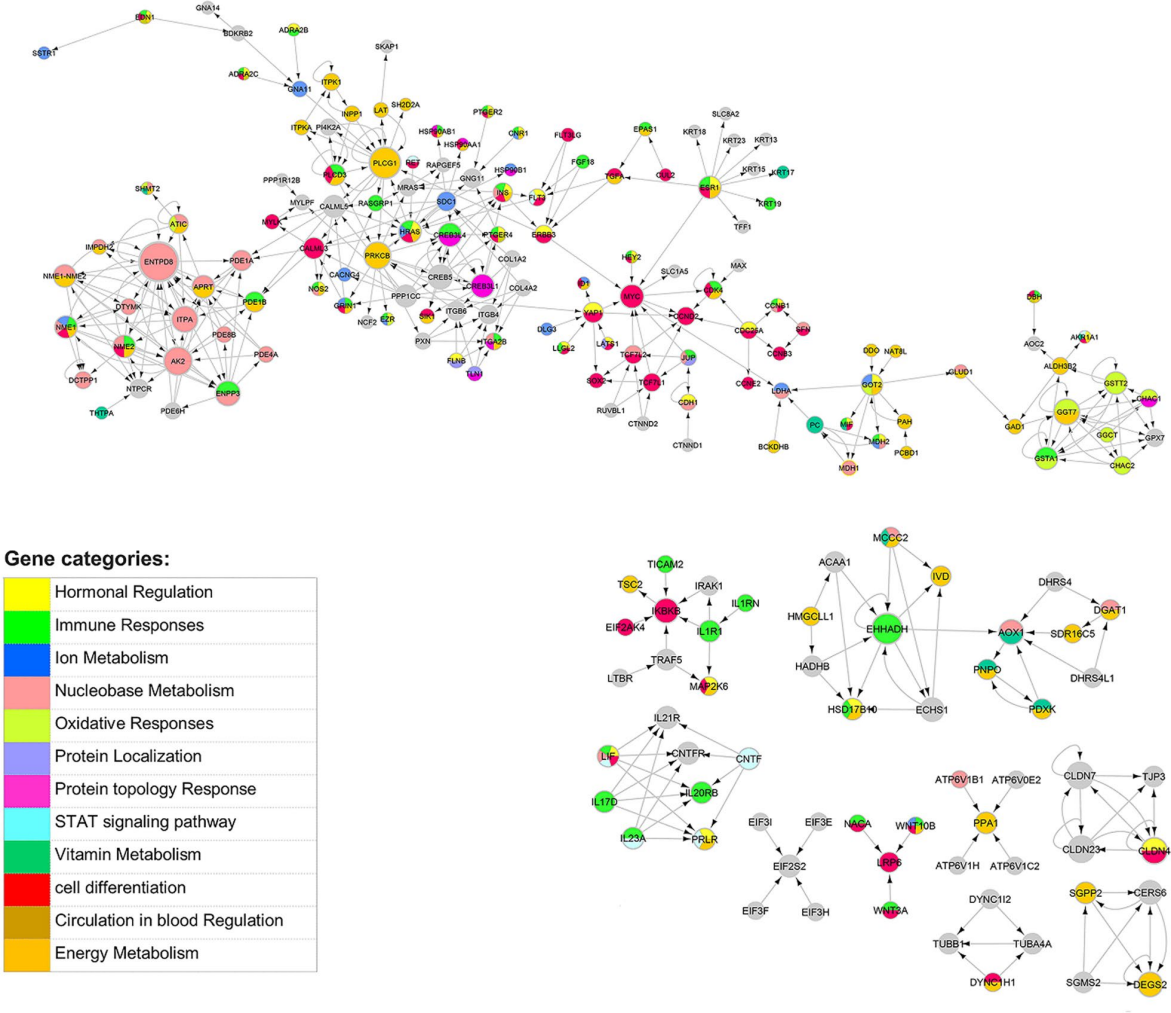
Signaling network of CTCs. The node size indicates the node degree. The direction indicates signaling among CTCs

The signaling crosstalk between two selected subnetworks as well as intermediate pathways between Immune and EMT was investigated by mapping them to the KEGG pathways and extracting induced subnetwork. A directed subnetwork of size 255 genes was extracted and illustrated in Fig. 3. The network density was 0.5 and it included 10 components, in which, *PLCG1* showed the highest betweenness value; and *MYC*, *MYLK*, and *MRAS* showed the highest closeness in the subnetwork.

To have a better illustration of the interplay among pathways, we categorized genes by colors based on ClueGO results. We detected 12 gene categories based on biological processes, including’Hormonal regulation’,’Immune responses’,’Ion metabolism’,’Nucleobase metabolism’,’Oxidative responses’,’Protein localization’,’Protein topology response’,’STAT singling pathway’,’Vitamin metabolism’,’Cell differentiation’,’Circulation in blood regulation’, and’Energy metabolism’ (Fig. 3). These categories were illustrated by colors on the subnetwork nodes, and the genes with no category remained grey. The nodes with multiple colors indicated different biological processes. The node size was illustrated by node degrees (Fig. 3). Genes *PLCG1* and *ENTPD8,* participated in ‘Energy Metabolism’ and ‘Nucleobase Metabolism’, were two hub nodes in our detected directed subnetwork. The ClueGO results were reported in Additional file 5.

### Distant metastasis classification model

The distant metastasis potential of two nominated subnetworks was assessed using SVM, neural network, and decision tree classification methods in GSE7390. The accuracy, sensitivity, and specificity scores of the SVM model for the EMT subnetwork were 79%, 78%, and 21%, respectively. The neural network accuracy, sensitivity, and specificity scores were 18%, 18%, and 80%, respectively. Eventually, the decision tree accuracy, sensitivity, and specificity scores were 60%, 60%, and 30%, respectively. These results refer to a full model (all genes in the subnetwork included). Comparing three models, the SVM model was the strongest method in classifying metastatic and non-metastatic patients, but the specificity score was too low.

The SVM accuracy, sensitivity, and specificity scores for immune-related subnetwork were 78%, 78%, and 78%, respectively. The neural network accuracy, sensitivity, and specificity scores were 85%, 85%, and 14%, respectively. Eventually, the decision tree accuracy, sensitivity, and specificity scores were 71%, 71%, and 36%, respectively. These results refer to a full model (all genes of subnetwork included). The specificity of the neural network and decision tree methods was low compared to the SVM model. Due to the results, the SVM model was the most powerful method in classifying metastatic and non-metastatic patients for the immune-related subnetwork. The SVM model accuracy, sensitivity, and specificity for the Immune subnetwork were superior to the EMT subnetwork.

The feature selection algorithms, for the SVM model, were implemented for both subnetworks. The WCC introduced 13 and 15 genes, and GA introduced 12, 17 genes for EMT- and immune-related subnetworks, respectively. The WCC introduced *HLA-E*, *MYLK*, *WIPF1*, *TLN1*, *F13A1*, *NRGN*, *ICAM2*, *PTGS1*, *SELP*, *PF4*, *ITGA2B*, *GFI1B*, *TUBB1*, *PTCRA*, *RUFY1*, *BIN2*, and *CLEC1B* and the GA introduced *CCL5*, *MYLK*, *WIPF1*, *TLN1*, *NRGN*, *GNG11*, *PTGS1*, *SELP*, *ITGA2B*, *MAX*, *GFI1B*, *P2RX1*, *PTCRA*, *RUFY1*, and *BIN2* for the immune subnetwork.

The SVM model, full model, for immune subnetwork validated in GSE9195. The accuracy, sensitivity, and specificity were 0.868; surprisingly, the validation scores were superior to GSE7390. The results confirmed that the immune-related genes detected in this study can classify metastatic and non-metastatic samples more precisely compared to the neural network and decision tree models, using two data sets. We implemented the classification methods to assess the metastasis potential of two nominated CTC-related subnetworks.

### Distant metastasis-free-survival and overall survival analyses

**Fig. 4:**
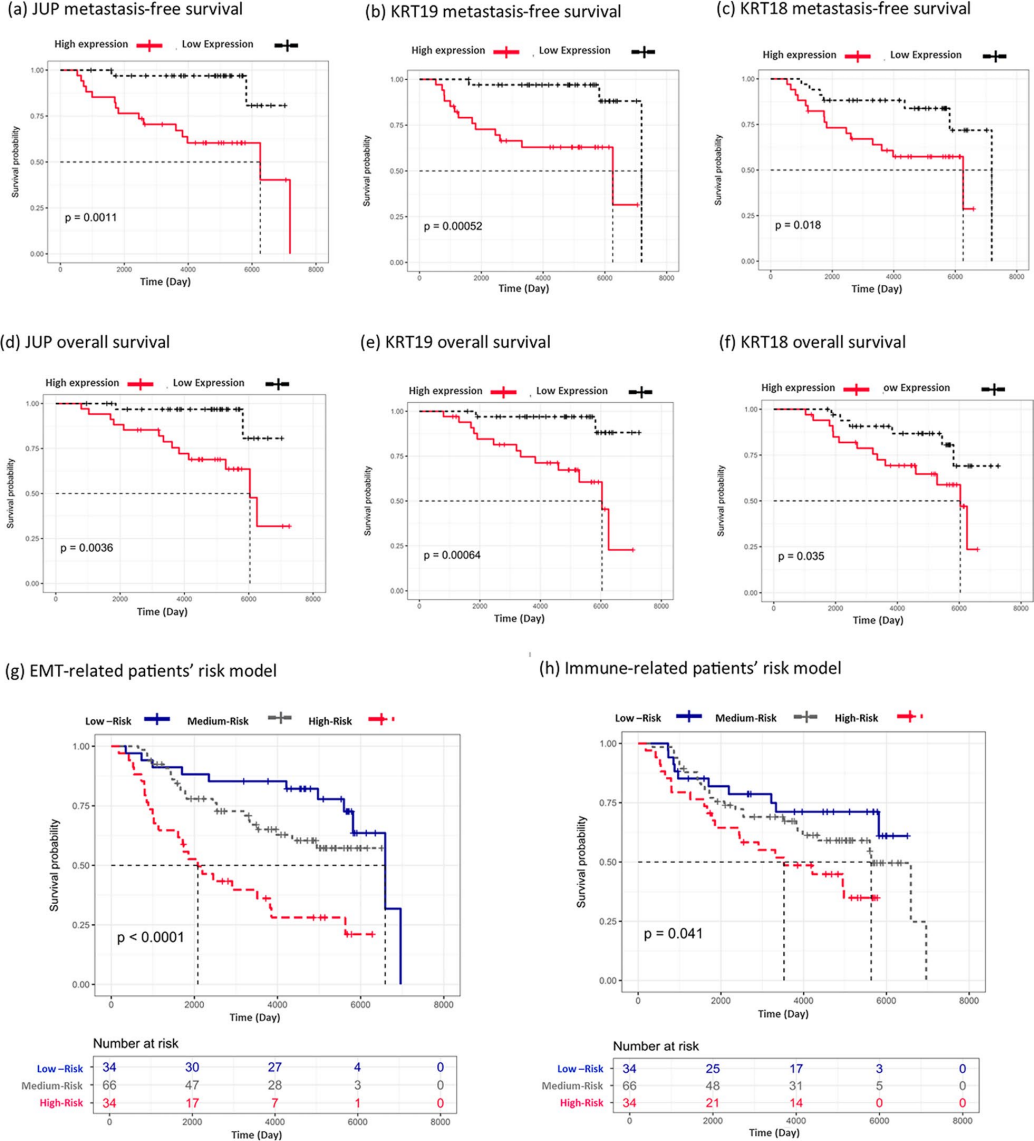
Metastasis free survival and overall survival. p indicate p-value of Log-Rnk test in **a**–**h** section. **g** a predictive metastasis risk model for EMT subnetwork. High risk indicates the upper-quartile of gene expression; low-risk indicates the lower-quartile of gene expression. **h** a predictive metastasis risk model for the immune subnetwork. High risk indicates the upper-quartile of gene expression; low-risk indicates the lower-quartile of gene expression

The association between gene expression and distant metastasis-free survival /overall survival was implemented to detect metastatic potential genes in two selected subnetworks. Overall survival and distant metastasis-free survival of *JUP*, *KRT18*, and *KRT19* were significant (Log-rank test p-value < 0.05; the exact p-values were reported in Figures) (Fig. 4a–f). These three genes belonged to the EMT subnetwork. The upregulation of *JUP*, *KRT18*, and *KRT19* was associated with more metastases; Therefore, the lower overall survival of patients (Fig. 4a–f). Moreover, *JUP*, *KRT18*, and *KRT19* were upregulated in CTC clusters (Fig. 1a). The lower distant metastasis-free survival and lower overall survival curves confirmed the importance of selected genes in metastasis. Therefore, they are important gene signatures in CTCs.

We fitted a metastasis-free-survival Cox-PH regression model for EMT and Immune subnetworks to assess patients’ metastasis risks through a predictive model. The Immune Cox-PH model included *RUFY1* and *P2RX1* variables (Likelihood ratio test p-value = 0.0295); and the EMT Cox-PH model included *RAN*, *PEBP1*, *KRT8*, *DSP*, *DDR1*, and *CLDN4* variables (Likelihood ratio test p-value = 0.0001016). The coefficients of variables and p-values were reported in Additional file 6 and Additional file 7. All the significant genes in the model had VIF < 2 to avoid multicollinearity problems, using the VIF-feature selection method (Additional file 8 and Additional file 9). The proportional hazard assumptions for two model variables were assessed by the Schoenfeld residuals (Additional file 10 and Additional file 11). The predictive Cox-PH models for distant metastasis-free survival for two subnetworks were illustrated in Fig. 4g, h. The concordance index, a performance evaluation measure, for EMT and Immune predictive Cox-PH models were 0.7 and 0.6, respectively. Therefore, the EMT model is more powerful in discriminating patients into low, medium, and high metastasis risk groups compared to the Immune model.

Of note, the concordance index is a generalization of the area under the ROC curve (AUC) that is modified for survival analysis. Concordance index can take into account censored data. Therefore, to evaluate more than one model, we use the concordance index that evaluates the candidate models' performance. The higher concordance index indicates more power in discrimination. The supplementary material was provided in Additional file 12.

## Discussion

Whereas multiple studies on circulating tumor cells (CTCs) as single CTCs or metastatic microemboli (CTC clusters) have been conducted, the molecular mechanisms of such rare cells are insufficiently characterized. CTCs bear several undiscovered metastatic potentials to overcome many restrictions, including extravasation of the primary tumor microenvironment, survival in the bloodstream, and successfully colonizing secondary organs; Therefore, a better understanding of the biological mechanisms of different types of CTCs, single/cluster, is essential.

This study was aimed to explore metastasis-related mechanisms within CTCs. We have implemented the co-expression analysis to detect subnetworks discriminating single/cluster CTCs (Fig. 1). Two of the subnetworks indicated a significant correlation to the trait (single/cluster status of CTCs). Our detected subnetworks illustrated immune- and EMT-related pathways (Fig. 2a, b). Due to previous studies, the immune-associated mechanisms and EMT pathways are of two major arms in breast cancer progression and metastasis, but investigating them in CTCs is not thoroughly studied [11].

First, we assess the EMT-related markers. To prepare cancer cells for migration and intravasation in the early stages of cancer, the keratin family, claudins, and cadherins must be downregulated through the EMT process in primary tumors; still, due to the surviving urgency of CTCs in the bloodstream and avoiding anoikis, a small number of tumor cells must be attached and break off from the primary site [29, 30]. These aggregated cells are called cluster CTCs. Therefore, cytoskeleton-related genes, such as the keratins, claudins, and cadherins should be upregulated in CTC clusters to survive shear forces in blood circulation. In our study, we identified a number of cytoskeleton-related genes that were reported in the EMT-related subnetwork. The plakoglobin (*JUP*), *KRT8*, *KRT18*, *KRT19*, CLDN4, and *EPCAM*, which their role in breast cancer metastasis was demonstrated in previous studies, are EMT markers [5, 30–33]. The *KRT8*, *KRT18*, *KRT19* are a group of cytoskeleton genes within the cellular cytoplasm called keratins. Although they are extensively used as diagnostic tumor markers, several studies have demonstrated their involvement in cancer cell invasion and metastasis as well as in treatment responsiveness [31, 33]. Keratins are the intermediate filament-forming proteins of epithelial cells that organize the internal three-dimensional cellular structure; In fact, they act in cell shape maintenance for bearing tensions. [33]. Moreover, the plakoglobin (*JUP*), the upregulation of which in breast cancer CTC clusters in comparison to single cells was demonstrated in Aceto, Nicola, et al. study, is one of the cell junction genes that hold tumor cells together to leave primary tumor as CTC clusters [5]. In our study, the overexpression of *JUP*, *KRT18*, and *KRT19* as well as the overall survival and metastasis-free survival were significant (Fig. 4a–f). Therefore, they may play an essential role in the integrity of CTC clusters in the bloodstream shear forces. Meanwhile, *EPCAM* and cytokeratins have been reported as detection markers in the enrichment of CTCs [34]. Of note, few markers guide scientists to detect metastatic patients in the clinic therefore the rest of the genes detected in the EMT subnetwork were not investigated in CTC studies; Consequently, they could be new targets in experimental studies for CTCs.

As we know the other key factor in metastasis is immune responses. Several types of immune cells ambiguously reveal anti- and pro-tumor behaviors [35]. The immunosuppressive microenvironment of tumors protects the primary tumor cells. Nevertheless, while tumor cells extravasate and enter circulation, they lose their tumor protection; Therefore, they must adapt themselves to escape immune surveillance [1, 35]. The interplay between immune cells and cytokeratins may contribute to evasion of CTCs from immune surveillance. For example, the cytotoxic T lymphocytes (CTLs) were recruited by recognizing tumor antigens presented by major histocompatibility class I (*MHCI*) [36, 37]. The under-expression of *MHCI* in tumor cell surface guides them to hide from CTLs and thereby survive in circulation. Moreover, the overexpression of cytokeratins such as *KRT8*, and together with heterodimeric partners *KRT18* and *KRT19* inhibit *MHCI* interactions with CTLs [35, 36]. All these findings, overexpression of *KRT8*, *KRT18*, *KRT19*, and under-expression of *HLA-E*, are consistent with our results which highlight the CTC cluster potential to evade the immune system; consequently, longer survival (Fig. 1a, b). Therefore, there might be an interplay between EMT and the immune system in the CTCs. On the contrary, Thangavel, Hariprasad, et al. did not find significant differential expression for EMT-related markers between CTC clusters and CTC singles [38]. Their study was implemented on basal-like breast cancer tumors in which a group of specific EMT markers was assessed while we analyzed the ER + data with a different method. In Thangavel, Hariprasad et al. Study, the EMT markers were selected using a scoring method introduces by George, Jason T., et al., while our EMT markers were detected using the co-expression and enrichments results (Fig. 2a) [39]. However, our results may indicate the strength of our study to identify the right markers in CTCs and do not violate former studies.

Of note, several studies supported the association between EMT and immune cell escape of cancer cells [40, 41]. Moreover, a plethora of genes and signals support stemness pathways such as Wnt, TGF-β, and NOTCH in CTCs [10]. Downregulation of *DAB2*, which is a putative tumor suppressor and involves in the TGF-β pathway and promotes EMT, was reported in breast cancer tumors [42, 43]. Therefore, the under-expression of *DAB2* in CTC clusters might be related to the stemness phenotype which helps CTC clusters to escape the immune system. In our study, *DAB2*, the expression of which was downregulated in CTC clusters (the logarithm of fold change = − 5.7), was detected in our immune subnetwork (Fig. 1b). Therefore, our findings may indicate the survival potential of CTC clusters in circulation, which were consistent with previous studies on cancer biology. Whereas the CTC clusters have higher metastatic potential due to less frequency in metastatic patients, but the single cells contribute metastasis either. Hereof, several studies such as Szczerba, Barbara Maria, et al. indicated more single-cell, about 88.0%, detected in metastatic patients [44]. Therefore, either single CTCs or CTC clusters have metastasis potential with different molecular mechanisms.

Not only the immune system and EMT but also the intermediate pathways are important in the progression of CTCs. The crosstalk between the signaling pathways of immune response and hormonal regulations, such as the fluctuation of menstrual cycles, was investigated by Atashgaran, Vahid, et al. in breast cancer [45]. Furthermore, they showed the dis-regulation of hormonal factors affecting genome instability and the decrease of immune surveillance in breast cancer [45]. The immune responses and hormonal regulation were detected in our study too (Fig. 3). On the other hand, we know EMT is a complex process through which tumor cells facilitate their dissemination and acquire stemness characteristics, or it is better to say that cells lose their differentiation [14, 46]; Therefore, bases on previous studies, not only signaling pathways of stemness but also the stimulation of self-renewing pathways in tumor cells are essential in tumorigenesis [14, 46]. Such several metastatic prone pathways and the interplay among all of them were detected in circulating tumor cells in our results (Figs. 2c and 3). Inasmuch as the CTCs are tumor cells that reflect characteristics of primary tumors and they also gain more additional propensity to survive in blood-stream, they were able to extravasate secondary site and metastasize. These pro-metastatic cells need to recruit different signaling pathways that interplay among them leads to creating multi-role cells that reflect great metastatic and survival potentials. As a result, characterizing multiple aspects of CTCs involved in cancer progression is essential; Moreover, it would be useful in finding novel biomarkers or patients’ treatment strategies.

## Conclusions

In summary, although CTCs, which are cancer-related biomarkers, are applied in the clinic, the molecular mechanisms were not investigated well. The unknown crosstalks among multiple pathways including EMT and immune responses improve the survival of CTCs in the patients’ blood. Therefore, they may contribute to therapeutic resistance and metastasis. Computational investigations on CTCs suggest novel metastatic biomarkers which could be new targets for experimental studies or therapeutic aims.

## Supplementary Information

**Additional file 1**. Midnightblue (immune) subnetwork. The list of genes, module membership, the logarithm of fold change, differential analysis. adj-p.value, and gene-significance statistics.

**Additional file 2**. Turquoise (EMT) subnetwork. The list of genes, module membership, the logarithm of fold change, differential analysis. adj-p.value, and gene-significance statistics.

**Additional file 3**. Correlation and p-values for subnetwork/trait. The metastasis-related subnetwork was selected, using subnetwork/trait correlation. ($\left|\mathrm{correlation }\right|$> 0.5).

**Additional file 4**. The preservation statistics. This table includes ${Z}_{summary }$ and ${Median}_{rank}$ statistics.

**Additional file 5**. Biological pathways in the directed network. We categorized genes of the detected directed network, using ClueGO plugin in Cytoscape. The Biological pathways and p-values were reported.

**Additional file 6**. The EMT subnetwork cox-PH results. The cox-PH analysis was implemented for the EMT subnetwork genes. The selected genes, coefficients, and p-values were reported.

**Additional file 7**. The Immune subnetwork cox-PH results. The cox-PH analysis was implemented for the immune subnetwork genes. The selected genes, coefficients, and p-values were reported.

**Additional file 8**. EMT VIF values. To investigate multicollinearity in the cox-PH model, we calculated the Variance Inflation Factor (VIF). The VIF < 10 indicates no multicollinearity. The VIF of immune genes was reported.

**Additional file 9**. Immune VIF values. To investigate multicollinearity in the cox-PH model, we calculated the Variance Inflation Factor (VIF). The VIF < 10 indicates no multicollinearity. The VIF of immune genes was reported.

**Additional file 10**. The Schoenfeld residuals for EMT genes. The proportional hazard ratio investigated, using the Schoenfeld residuals. The residuals (red dots) must be between the curves.

**Additional file 11**. The Schoenfeld residuals for immune genes. The proportional hazard ratio investigated, using the Schoenfeld residuals. The residuals (red dots) must be between the curves.

**Additional file 12**. Supplementary methods.

## Data Availability

The public data were used in this article including GSE51827, GSE9195, GSE7390, and GSE86978.

## Abbreviations

CTC: Circulating tumor cell
EMT: Epithelial–Mesenchymal-Transition
SVM: Support vector machine
PTCRA: Pre T Cell Antigen Receptor Alpha
F13A1: Coagulation Factor XIII A Chain
LAT: Linker For Activation Of T Cells
ICAM2: Intercellular Adhesion Molecule 2
OS: Overall survival
PCA: Principal component analysis
WCC: World competitive contest
GA: Genetic algorithm
ANN: Artificial neural network
Cox-PH: Cox proportional hazard ratio
DEG: Differentially expressed gene
VIF: Variance Inflation Factor
FDR: False discovery rate
