# Supplementary figures and images for: Involvement of immune system and Epithelial–Mesenchymal-Transition in increased invasiveness of clustered circulatory tumor cells in breast cancer
Source: BMC Med Genomics. 2021 Nov 20;14:273. doi: 10.1186/s12920-021-01112-9 (PMC8605524; doi:10.1186/s12920-021-01112-9)

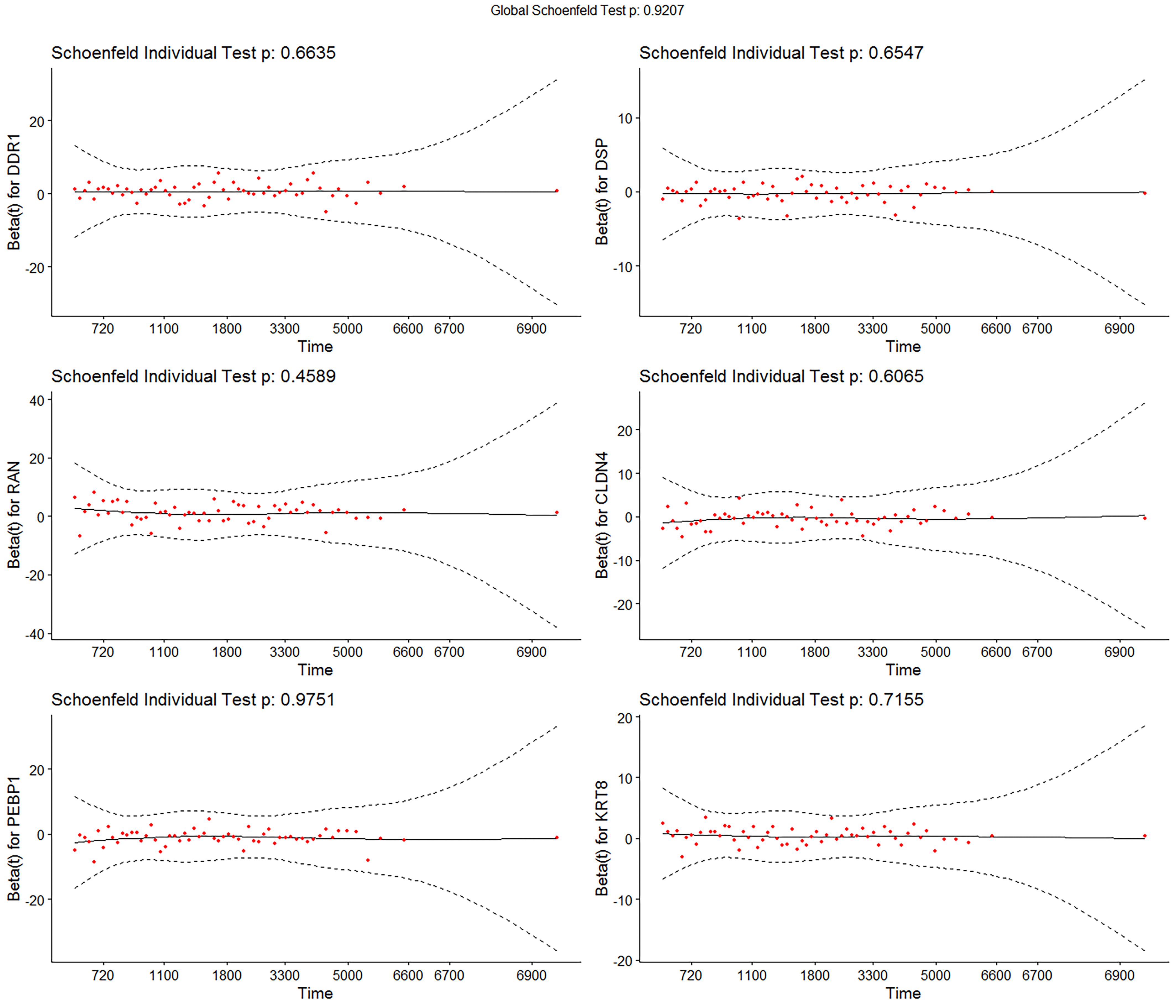

Supplement: Supplementary file 10 — Additional file 10. The Schoenfeld residuals for EMT genes. The proportional hazard ratio investigated, using the Schoenfeld residuals. The residuals (red dots) must be between the curves. [file 12920_2021_1112_MOESM10_ESM.tif]

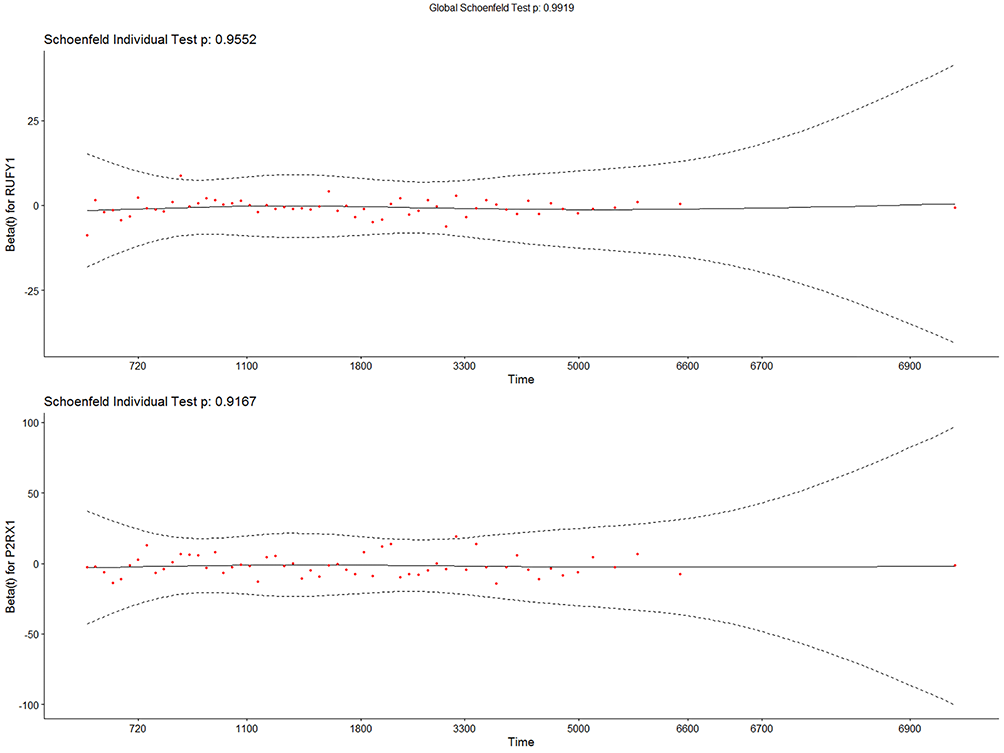

Supplement: Supplementary file 11 — Additional file 11. The Schoenfeld residuals for immune genes. The proportional hazard ratio investigated, using the Schoenfeld residuals. The residuals (red dots) must be between the curves. [file 12920_2021_1112_MOESM11_ESM.tiff]
