# Supplementary material for: Involvement of immune system and Epithelial–Mesenchymal-Transition in increased invasiveness of clustered circulatory tumor cells in breast cancer
Source: BMC Med Genomics. 2021 Nov 20;14:273. doi: 10.1186/s12920-021-01112-9 (PMC8605524; doi:10.1186/s12920-021-01112-9)
Supplement: Supplementary file 12 — Additional file 12. Supplementary methods. [file 12920_2021_1112_MOESM12_ESM.docx]

SUPPLEMENTARY METHODS

We included the detailed scripts used in this study in the following section.

## 1 | supplementary scripts

The following scripts were used for quality control, pre-processing, and normalization of CTC datasets (GSE86978 and GSE51827).

#-------------------------------

# Reading data

#--------------------------------

all.counts <- read.csv("/GSE86978_readCounts.csv")

dim(all.counts)

rownames(all.counts) <-all.counts$X

all.counts$X <- NULL

info_count <-read.csv("/my Data/gse86978 info .csv")

dim(info_count)

info_count<-read.csv("my Data/gse86978 info 1.csv")

mer <- paste(a$sample,a$status,sep = "..")

dim(all.counts)

#------------------------------------------------------

# Filtering out low-abundance genes

#------------------------------------------------------

all.counts <- na.omit(all.counts)

all.counts <- all.counts[rowSums(all.counts)>0,]

dim(all.counts)

#----------------------------------------------------

# create single cell object

#----------------------------------------------------

sce <- SingleCellExperiment(assays = list(counts =as.matrix(all.counts)),colData = info_count)

sce <- calculateQCMetrics(sce,use_spikes = FALSE)

colnames(colData(sce))

libsize.drop <- isOutlier(sce$total_counts, nmads=3, type="lower", log=TRUE)

feature.drop <- isOutlier(sce$total_features_by_counts, nmads=3, type="lower", log=TRUE)

sce <- sce[,!(libsize.drop | feature.drop )]

data.frame(ByLibSize=sum(libsize.drop), ByFeature=sum(feature.drop),

Remaining=ncol(sce))

dim(sce)

# mean filter

ave.counts <- rowMeans(counts(sce))

keep <- ave.counts >= .5

sum(keep)

#-----------------------------------------------------

# Normalization of cell-specific biases

#-----------------------------------------------------

sce <- computeSumFactors(sce, sizes=c(60, 65, 70, 74))

summary(sizeFactors(sce))

sce <- normalize(sce)

# extract Normalized expression

log_norm_data<-logcounts(sce)

dim(log_norm_data)
